# Quantification of Tau Load Using [^18^F]AV1451 PET

**DOI:** 10.1007/s11307-017-1080-z

**Published:** 2017-04-03

**Authors:** Sandeep S. V. Golla, Tessa Timmers, Rik Ossenkoppele, Colin Groot, Sander Verfaillie, Philip Scheltens, Wiesje M. van der Flier, Lothar Schwarte, Mark A. Mintun, Michael Devous, Robert C. Schuit, Albert D. Windhorst, Adriaan A. Lammertsma, Ronald Boellaard, Bart N. M. van Berckel, Maqsood Yaqub

**Affiliations:** 1 Department of Radiology and Nuclear Medicine, VU University Medical Center, Amsterdam, Netherlands; 2 Alzheimer Center & Department of Neurology, VU University Medical Center, Amsterdam, Netherlands; 3 Department of Epidemiology & Biostatistics, VU University Medical Center, Amsterdam, Netherlands; 4 Department of Anaesthesiology, VU University Medical center, Amsterdam, Netherlands; 5 Avid Radiopharmaceuticals, Inc., Philadelphia, USA; 6 Department of Nuclear Medicine & Molecular Imaging, University of Groningen, University Medical Center Groningen, Groningen, The Netherlands

**Keywords:** [^18^F]AV1451, PET Pharmacokinetic Modeling, Flortaucipir, Alzheimer’s Disease, Tau Imaging

## Abstract

**Purpose:**

The tau tracer [^18^F]AV1451, also known as flortaucipir, is a promising ligand for imaging tau accumulation in Alzheimer’s disease (AD). Most of the previous studies have quantified tau load using standardized uptake value ratios (SUVr) derived from a static [^18^F]AV1451 scan. SUVr may, however, be flow dependent and, especially for longitudinal studies, should be validated against a fully quantitative approach. The objective of this study was to identify the optimal tracer kinetic model for measuring tau load using [^18^F]AV1451.

**Procedures:**

Following intravenous injection of 225 ± 16 MBq [^18^F]AV1451, 130 min dynamic PET scans were performed in five biomarker confirmed AD patients and five controls. Arterial blood sampling was performed to obtain a metabolite-corrected plasma input function. Next, regional time–activity curves were generated using PVElab software. These curves were analysed using several pharmacokinetic models.

**Results:**

The reversible single tissue compartment model (1T2k_V_B_) was the preferred model for all but one control. For AD patients, however, model preference shifted towards a reversible two tissue compartmental model (2T4k_V_B_). The simplified reference tissue model (SRTM) derived binding potential (BP_ND_) showed good correlation (AD: *r* ^2^ = 0.87, slope = 1.06; controls: *r* ^2^ = 0.87, slope = 0.86) with indirect plasma input binding (distribution volume ratio-1). Standardized uptake value ratios (80–100 min) correlated well with DVR (*r* ^2^ = 0.93, slope = 1.07) and SRTM-derived BP_ND_ (*r* ^2^ = 0.84, slope = 0.95). In addition, regional differences in tracer binding between subject groups in different tau-specific regions were observed.

**Conclusions:**

Model preference of [^18^F]AV1451 appears to depend on subject status and, in particular, V_T_. The relationship between model preference and V_T_ suggests that (higher) tau load may be reflected by a second tissue compartment. Nevertheless, consistent results can be obtained using a 2T4k_V_B_ model. In addition, SRTM can be used to derive BP_ND_.

**Electronic supplementary material:**

The online version of this article (doi:10.1007/s11307-017-1080-z) contains supplementary material, which is available to authorized users.

## Introduction

A core neuropathological hallmark of Alzheimer’s disease (AD) is the aggregation of hyperphosphorylated tau proteins, assembling into neurofibrillary tangles [1]. Neuropathologically, animal and cerebrospinal fluid studies have shown that increased tau load is strongly related to more synaptic loss [2] and poorer cognitive performance [3]. The novel positron emission tomography (PET) tracer [^18^F]AV1451, also known as flortaucipir, has been developed to visualize tau pathology in the living human brain. *In vitro* [^18^F]AV1451 binds with high affinity to paired helical filaments of tau [4–7]. In addition, it has been demonstrated *in vivo* that the degree of [^18^F]AV1451 uptake corresponds with disease severity [8–10] and that its distribution follows the prototypical spatial pattern described by Braak and Braak [1, 11].

In most of the previous studies, [^18^F]AV1451 uptake has typically been measured using semi-quantitative methods, such as the standard uptake value ratio (SUVr) [5, 8, 9]. SUVr has several advantages, such as shorter scan duration, reduced likelihood of patient movement and computational simplicity [12]. On the other hand, SUVr is also based on two important assumptions. Firstly, it is assumed that the tracer is in equilibrium, *i.e.,* the ratio of specific to non-specific uptake is constant. Secondly, it is assumed that there is no specific tracer binding in the reference region. Before simplified models can be used, these underlying assumptions need to be validated, especially in AD with its progressive decrease in cerebral blood flow (CBF). This decrease in CBF is not the same for all brain regions, as the cerebral cortex is more affected than the cerebellum [13]. Consequently, differences in tracer delivery between target (cortex) and reference (cerebellum) brain areas will change over time due to progression of disease. Indeed, it has been shown that SUVr may provide biased information in longitudinal amyloid studies [14].

The main objective of the present study was to identify the optimal tracer kinetic model for quantifying tau load using [^18^F]AV1451. A second objective was to assess the validity of the simplified reference tissue model. Finally, as data were acquired in both controls and AD patients, a preliminary comparison between subject groups was performed.

## Methods

### Participants

Five cognitively normal controls and five patients with probable AD from the Amsterdam Dementia Cohort of the VU University Medical Center were included. All subjects underwent standardized dementia screening, including medical history, neurological examination, neuropsychological testing, laboratory tests, brain magnetic resonance imaging (MRI) and a lumbar puncture to quantify Aβ-42, total tau and phosphorylated tau in cerebrospinal fluid (CSF) [15]. A consensus diagnosis was obtained during a multidisciplinary meeting using established diagnostic criteria [16]. AD patients met diagnostic criteria for probable AD with at least intermediate likelihood due to a positive [^18^F]florbetaben amyloid PET scan (visual read) and/or CSF profile (Aβ_42_ < 550 pg/ml, tau >375 pg/ml, p-tau >52 pg/ml) [16, 17]. Controls tested within normal limits at neuropsychological examination and clinical examination. Exclusion criteria for all subjects were clinically significant cardiovascular disease or abnormalities on screening ECG, structural abnormalities on MRI that were likely to interfere with interpretation of PET and haemoglobin levels ≤8 in males and ≤7 in females. All subjects signed an informed consent, and the study was approved by the Medical Ethics Review Committee of the VU University Medical Center.

### Radiochemical Synthesis

[^18^F]AV1451 was produced on a NEPTIS radiosynthesizer (Ora, Philippeville, Belgium) according to Supplementary Fig. 1 starting from AV1622 (supplied by AVID, Philadelphia, PA, USA). Cyclotron (IBA Cyclone 18/9, IBA, Louvain-la-Neuve, Belgium) produced [^18^F]fluoride was trapped from the enriched water by solid-phase extraction on a Sep-Pak® Light Accell™ Plus (QMA) (Waters, Milford, MA, USA) and eluted from the QMA with 0.8 ml of a mixture of 50:50 water for injection (Braun, Oss, The Netherlands)/acetonitrile containing Kryptofix[2.2.2] (7 mg, 19 μmol) and potassium carbonate (0.75 mg, 5 μmol). After drying of the [^18^F]fluoride and Kryptofix/potassium carbonate, AV-1622 (1.5 mg, 2.6 μmol) in 2.0 ml of dimethyl sulphoxide was added and the reaction mixture was heated for 5 min at 110 °C followed by deprotection using 1 ml of 3 M hydrochloric acid at 100 °C for 5 min. The reaction mixture was then cooled to 50 °C and neutralized with 7 ml of 0.5 M NaOH in water for injection. The resulting solution was passed through an Oasis HLB Light cartridge (Waters), and the cartridge was washed with 5 ml of water for injection and the [^18^F]AV1451 was subsequently eluted with 1.5 ml of acetonitrile. The acetonitrile solution, containing the product [^18^F]AV1451, was subjected to HPLC purification (Zorbax Eclipse XDB-C18, 9.4 × 250 mm, 5 μm; eluent: 60:40 10 mM ammonium acetate in water/acetonitrile at a flowrate of 4 ml/min). The product was eluted at 8.5 min and was collected in 30 ml of water for injection, and the total solution was passed over a Sep-Pak® Light C18 cartridge (Waters). The cartridge was washed with 5 ml of water for injection, and the product was eluted with 1 ml of sterile ethanol followed by 2 ml of sterile saline into a sterile vial containing 7 ml of sterile saline and filtered over a sterile Millex GV PVDF 0.22 μm filter.

### Metabolite Analysis

Blood was collected in a heparin tube and centrifuged for 5 min at 5000 rpm. Plasma was separated from blood cells, and about 1 ml was diluted with 2 ml of 0.1 M hydrochoric acid and loaded onto a tC18 Sep-Pak cartridge, which was pre-activated by elution with 6 ml of methanol and 12 ml of water, respectively. The cartridge was washed with 3 ml water to collect the polar radioactive fraction. Thereafter, the tC18 Sep-Pak cartridge was eluted with 1 ml of methanol and 2 ml of water to collect the mixture of non-polar metabolites. This fraction was further analysed by HPLC on a Dionex Ultimate 3000 system (Dionex, Sunnyvale, CA, USA) and equipped with a 1-ml loop. As a stationary phase, a Phenonenex Gemini C18, 250 × 10 mm, 5 μm (Phenomenex, Torrance, CA, USA) was used. The mobile phase consisted of 75 % 0.1 % trifluoroacetic acid in water in acetonitrile. The eluent was collected with a fraction collector (Teledyne ISCO Foxy Jr., Lincoln, NE, USA), and the fractions were counted for radioactivity with a Wallac 1470 gamma counter (Perkin Elmer, Waltham, MA, USA).

### PET

PET scans were performed using a Gemini TF-64 PET/CT scanner (Philips Medical Systems, Best, The Netherlands). All subjects received a venous cannula for tracer injection and a radial artery cannula for arterial sampling. Head movements were restricted by a head holder with band and regularly checked during scanning. The scan protocol started with a low-dose CT for attenuation correction, followed by a 225 ± 16 MBq [^18^F]AV1451 injection. Simultaneously with tracer injection, a 60-min dynamic emission scan was initiated. After a 20-min break and following a second low-dose CT, an additional dynamic emission scan was performed during the interval 80–130 min post-injection. PET scans were reconstructed using 3D RAMLA with a matrix size of 128 × 128 × 90 and a final voxel size of 2 × 2 × 2 mm^3^. All standard corrections for dead time, decay, attenuation, randoms and scatter were performed. Both scan sessions were co-registered into a single dataset of 29 frames (1 × 15, 3 × 5, 3 × 10, 4 × 60, 2 × 150, 2 × 300, 4 × 600 and 10 × 300 s), in which the last 10 frames belonged to the second PET session.

During the first 60 min post-injection (p.i.), arterial blood was sampled continuously using an online detection system at a rate of 5 ml/min for the first 5 min and a rate of 2.5 ml/min thereafter [18]. In additional, manual arterial samples (8 ml) were withdrawn at set time points (5, 10, 15, 20, 40, 60, 80, 105 and 130 min p.i.). These samples were used to correct the whole blood TAC for plasma-to-whole blood ratios and for radiolabelled metabolites using established procedures. In addition, a correction for time delay was performed to obtain a metabolite-corrected arterial plasma input function.

### Image Analysis

All subjects underwent structural 3D–T1 weighted MRI on a 3.0 Tesla camera (Ingenuity TF PET/MR, Philips Medical Systems, Best, The Netherlands), including sagittal T1 weighted sequences. These T1 weighted MR images were co-registered to PET. MR images were segmented automatically into grey matter, white matter and CSF using SPM8 (Wellcome Trust Centre for Neuroimaging) incorporated in the PVElab software [19]. In addition, regions of interests (ROIs), as defined by the Hammers template [20], were delineated on the co-registered MRI scans. By projecting those ROIs onto the dynamic PET frames, regional time-activity curves (TACs) were generated.

### Kinetic Analyses

The ROI TACs (grey matter) were fitted using various models [21], and the metabolite corrected plasma input function. The compartmental models evaluated were standard reversible single tissue (1T2k), and reversible (2T4k) and irreversible (2T3k) two tissue compartmental models, all with and without blood volume fraction (V_B_) as an additional fit parameter. Standard non-linear regression fitting to these models was performed for each ROI. The optimal kinetic model for describing *in vivo* kinetics of [^18^F]AV1451 was selected based on the Akaike criterion (AIC) [22]. Macroparameters, such as binding potential (BP_ND_), volume of distribution (V_T_) and distribution volume ratio (DVR = ratio of target to cerebellar grey matter V_T_), were calculated for the model(s) of interest.

Performance of the simplified reference tissue model (SRTM) [23] with grey matter cerebellum as reference region was evaluated. Validation of SRTM was performed by comparing DVR derived from the optimal tracer kinetic plasma input model with SRTM-derived BP_ND_.

In addition, data were analysed using SUVr for the time interval 80–100 min after injection. Again, grey matter cerebellum was used as reference region. Resulting SUVr values were compared with DVR derived from the optimal tracer kinetic plasma input model and BP_ND_ derived from SRTM.

Finally, as a preliminary and exploratory assessment of disease-specific kinetics, V_T_ and BP_ND_ were compared between controls and AD patients. Based on previous [^18^F]AV1451 studies [9, 11], the following grey matter ROIs were selected for this comparison: (1) AD-specific regions (*i.e.,* bilateral hippocampus, inferior and middle temporal lobe and posterior cingulate gyrus), (2) areas that are susceptible to off-target binding (basal ganglia), (3) a region that is often used as reference region (cerebellum) and (4) whole brain grey matter.

## Results

### Participants

Five controls with an average age of 67.8 ± 5.5 years and an MMSE score of 29.0 ± 0.7 were included. In addition, 5 AD patients with an average age of 64.6 ± 8.9 years, an MMSE score of 23.4 ± 4.3 and a disease duration of 2.3 ± 1.1 years were included. There was no significant difference in age between AD patients and controls. All AD subjects were amyloid positive (on Florbetaben PET and/or CSF markers). Three controls were amyloid negative, whereas two subject had a positive Florbetaben PET scan.

### [^18^F]AV1451 Production

[^18^F]AV1451 was obtained in 16–30 % uncorrected yield (9–13 GBq) as a sterile, isotonic and pyrogen-free solution with a specific activity of 93–272 GBq/μmol at end of synthesis in >99 % radiochemical purity.

### Blood Analysis

**Fig. 1:**
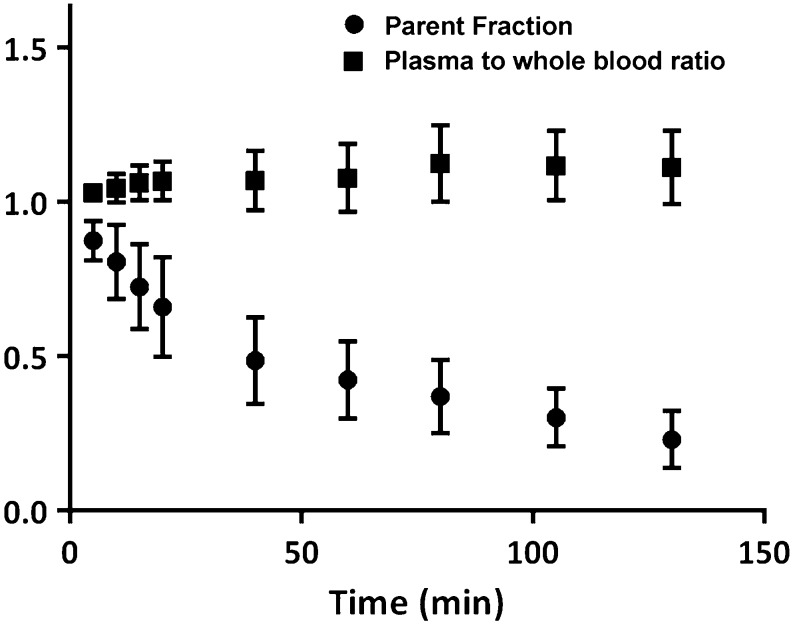
Mean (±SD) of the plasma-to-whole blood ratio and the parent [18F]AV1451 fraction in plasma for all 10 subjects.

Considerable metabolism was seen with 23 ± 9 % parent tracer left at 130 min p.i. Nevertheless, robust estimation of parent fractions was possible for all time points, and no significant difference between these fractions was seen between subject groups. For all subjects, the measured plasma-to-whole blood ratio was rather constant throughout the entire scan duration. Fig. 1 illustrates the mean plasma-to-whole blood ratio and parent fraction as function of time for all individuals.

### Tracer Kinetics

**Fig. 2:**
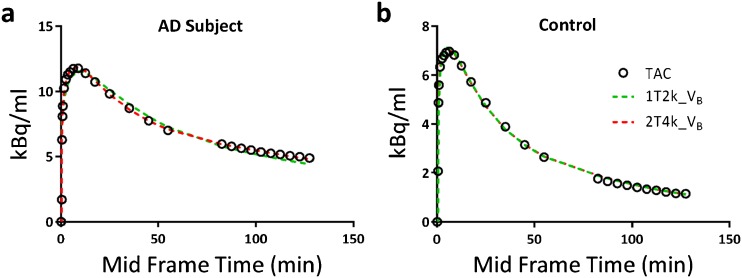
1T2k_V_B_ and 2T4k_V_B_ model fits through whole brain grey matter TACs of **a** an AD patient and **b** a control.

**Fig. 3:**
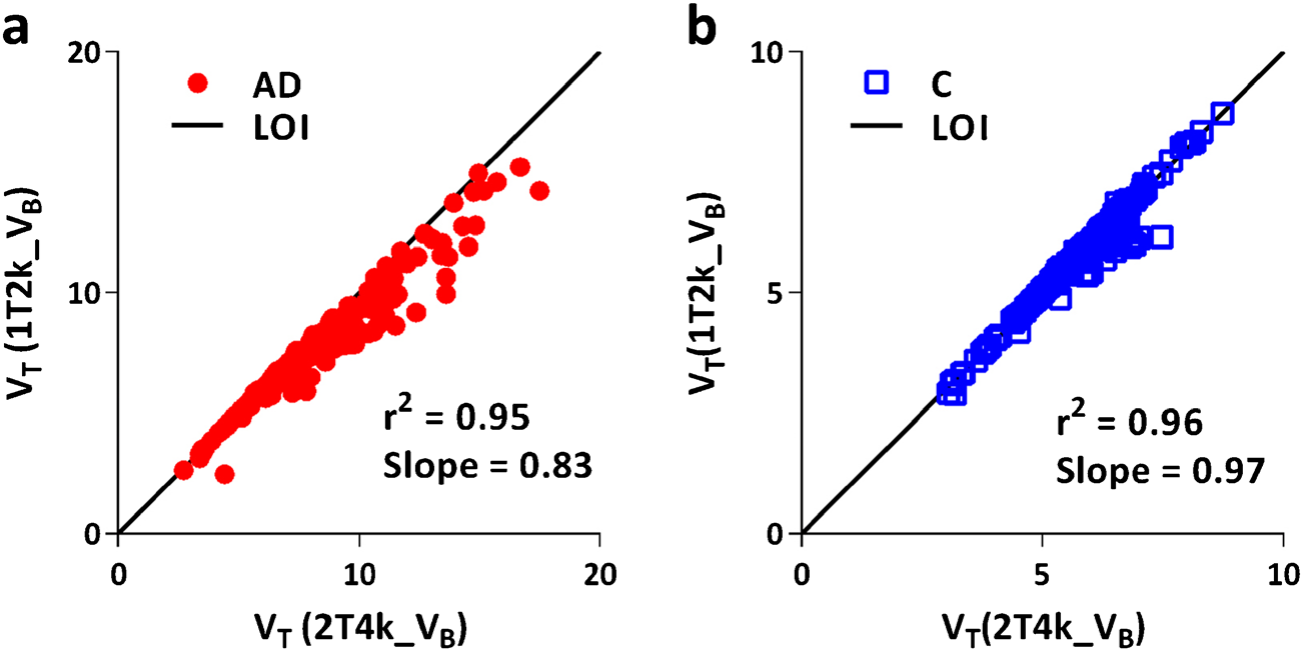
Comparison of volumes of distribution (V_T_) estimated using 2T4k_V_B_ and 1T2k_V_B_ models for **a** AD patients and **b** controls.

**Fig. 4:**
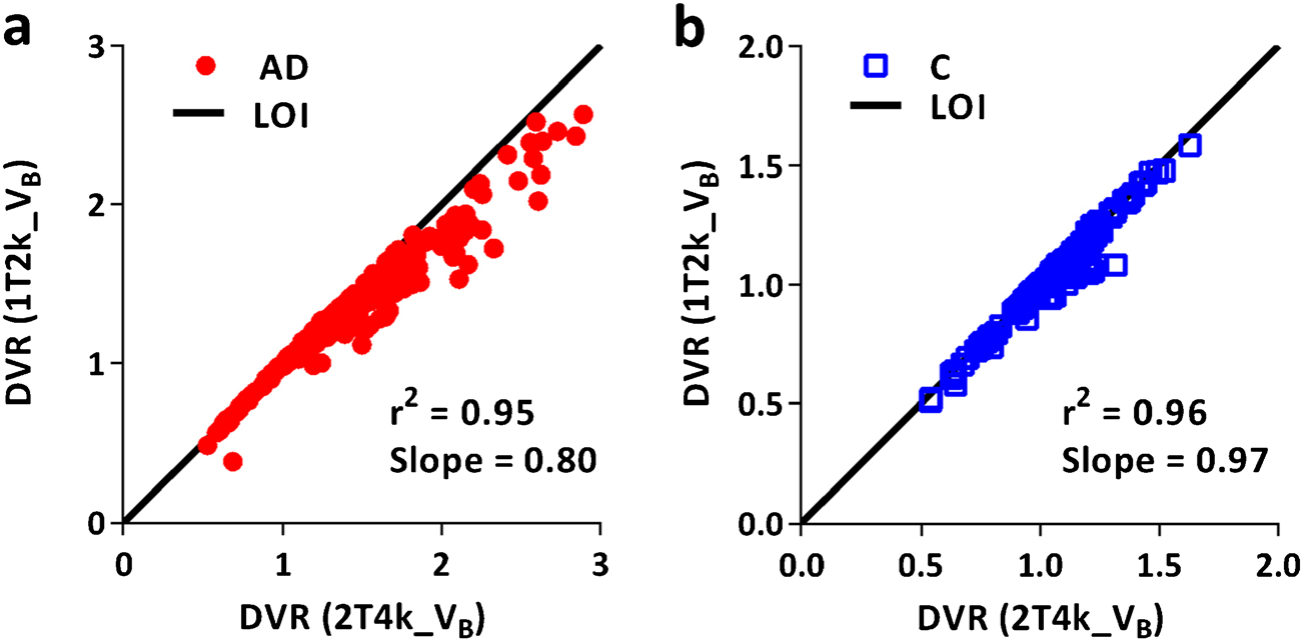
Comparison of distribution volume ratios (DVR) estimated using 2T4k_V_B_ and 1T2k_V_B_ models for **a** AD patients and **b** controls.

Model fits for all ROIs (grey matter) were assessed according to the AIC criterion. Fig. 2 illustrates model fits through a whole brain TACs of a typical AD patient and a control. All data from the AD patients were best fitted using a 2T4k_V_B_ model, but tracer kinetics in all but one controls were best described by a 1T2k_V_B_ model. Macroparameters (V_T_, DVR) derived from the two preferred models (2T4k_V_B_ and 1T2k_V_B_) were compared with each other to assess the impact of model preference on parameter estimation. Scatter plots of V_T_ estimated from the two models are shown in Fig. 3. Scatter plots of DVR obtained from the two models for both subject groups are presented in Fig. 4. In AD patients, directly derived BP_ND_ (=k_3_/k_4_) values estimated using the 2T4k_V_B_ model did not correlate with DVR-1 (*r* ^2^ = 0.02, slope = −0.03), indicating that direct estimation of BP_ND_ suffers from a high degree of imprecision. This was even more pronounced for controls, where direct estimation of BP_ND_ was not possible due to very high standard errors.

### SRTM

**Fig. 5:**
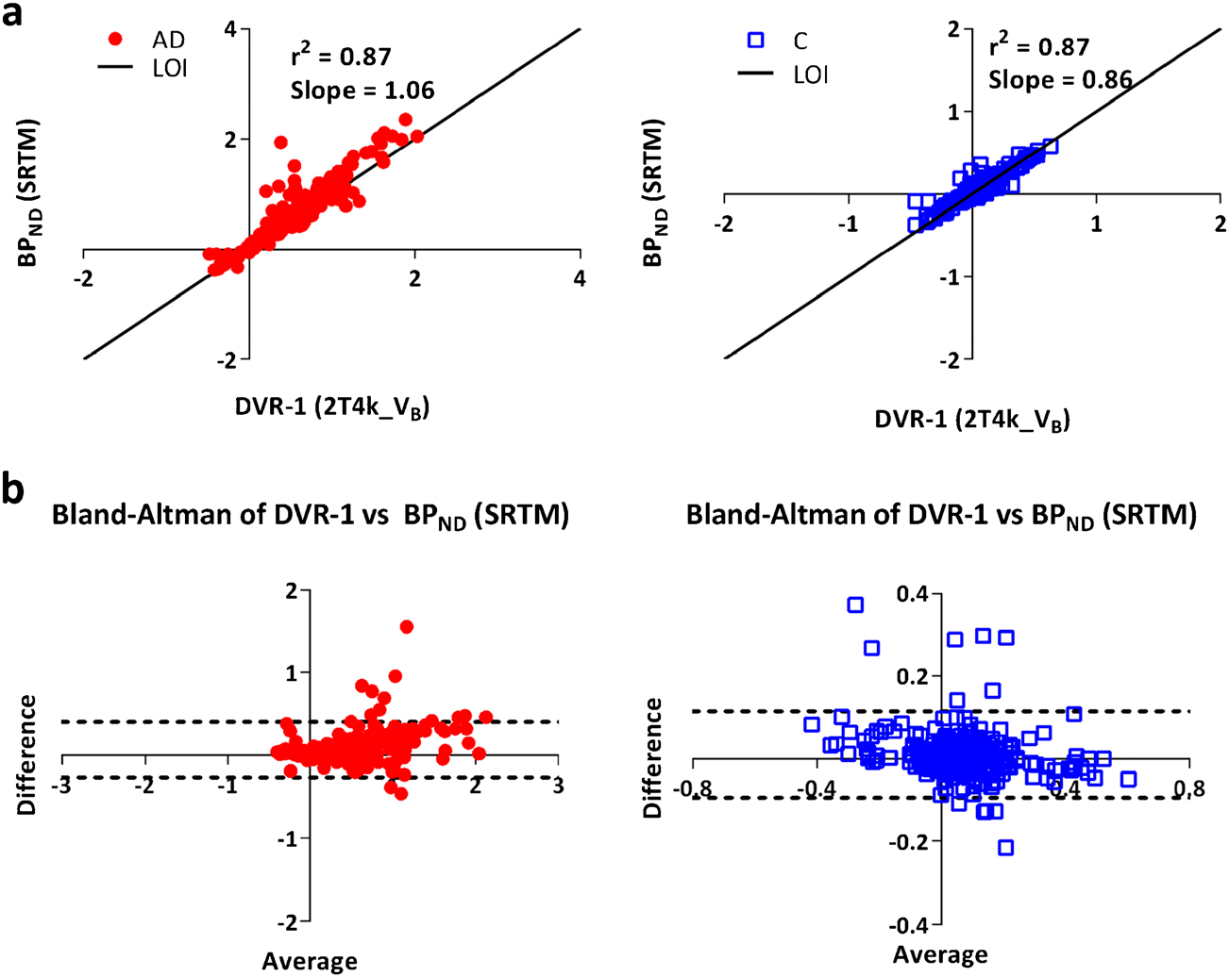
**a** Scatter plots and **b** Bland-Altman plots for the relationship between SRTM-derived BP_ND_ and DVR-1 derived from the 2T4k_V_B_ model for both AD patients and controls.

For SRTM, grey matter cerebellum was used as reference region. Fig. 5 shows the comparison of regional SRTM-derived BP_ND_ with DVR-1 obtained using the 2T4k_V_B_ model across all grey matter ROIs investigated.

### SUVr

**Fig. 6:**
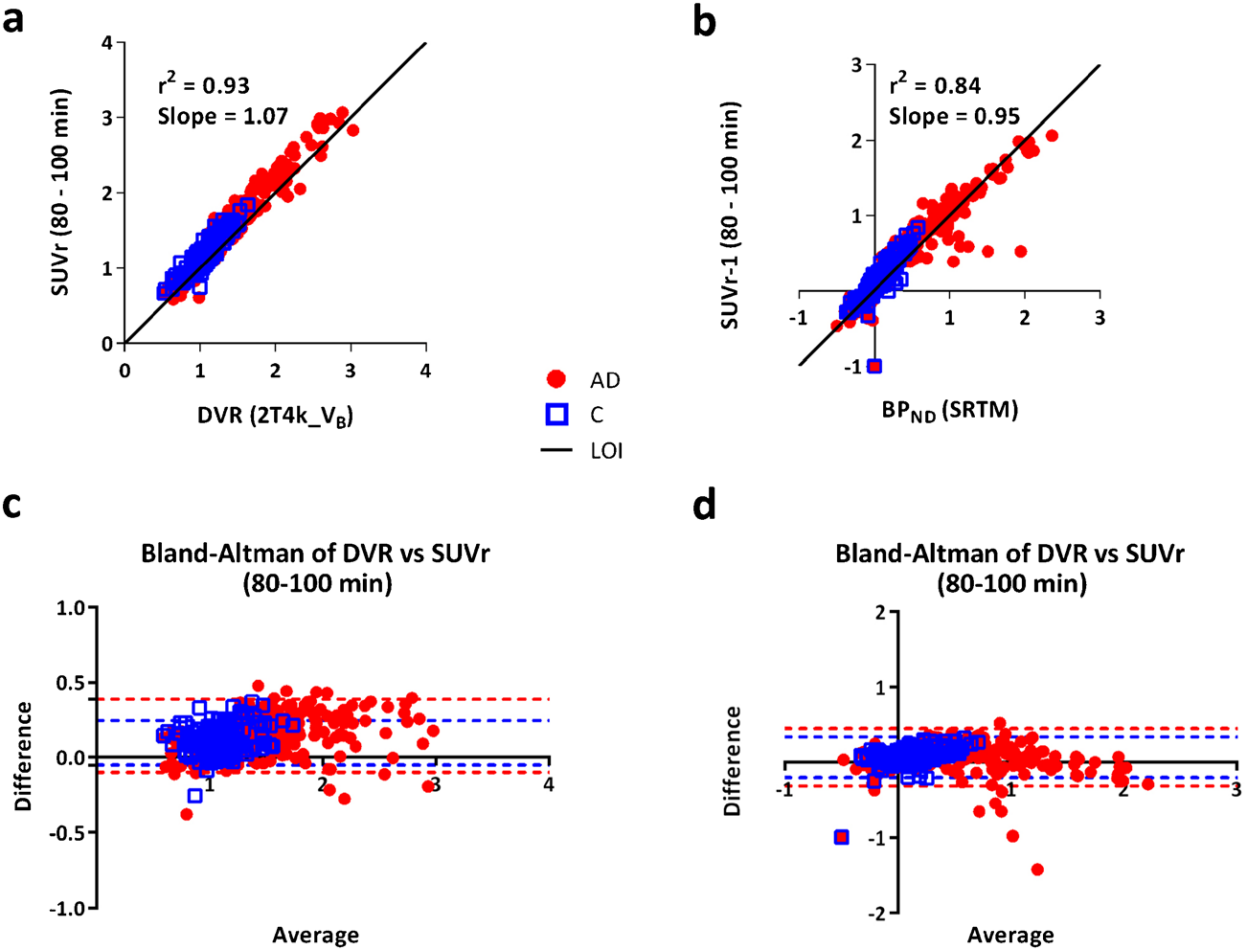
Scatter plots and Bland-Altman plots comparing SUVr derived from the 80–100 min scan interval to DVR derived from 2T4k_V_B_ (**a**, **c**) and SRTM-derived BP_ND_ (**b**, **d**).

Comparisons of SUVr (80–100 min) with DVR derived from the 2T4K_V_B_ model and BP_ND_ derived from SRTM are shown in Fig. 6. High correlations between SUVr and DVR (*r* ^2^ = 0.93, slope = 1.07) and between SUVr-1 and BP_ND_ (*r* ^2^ = 0.84, slope = 0.95) were obtained.

### AD Patients *Versus* Controls

**Fig. 7:**
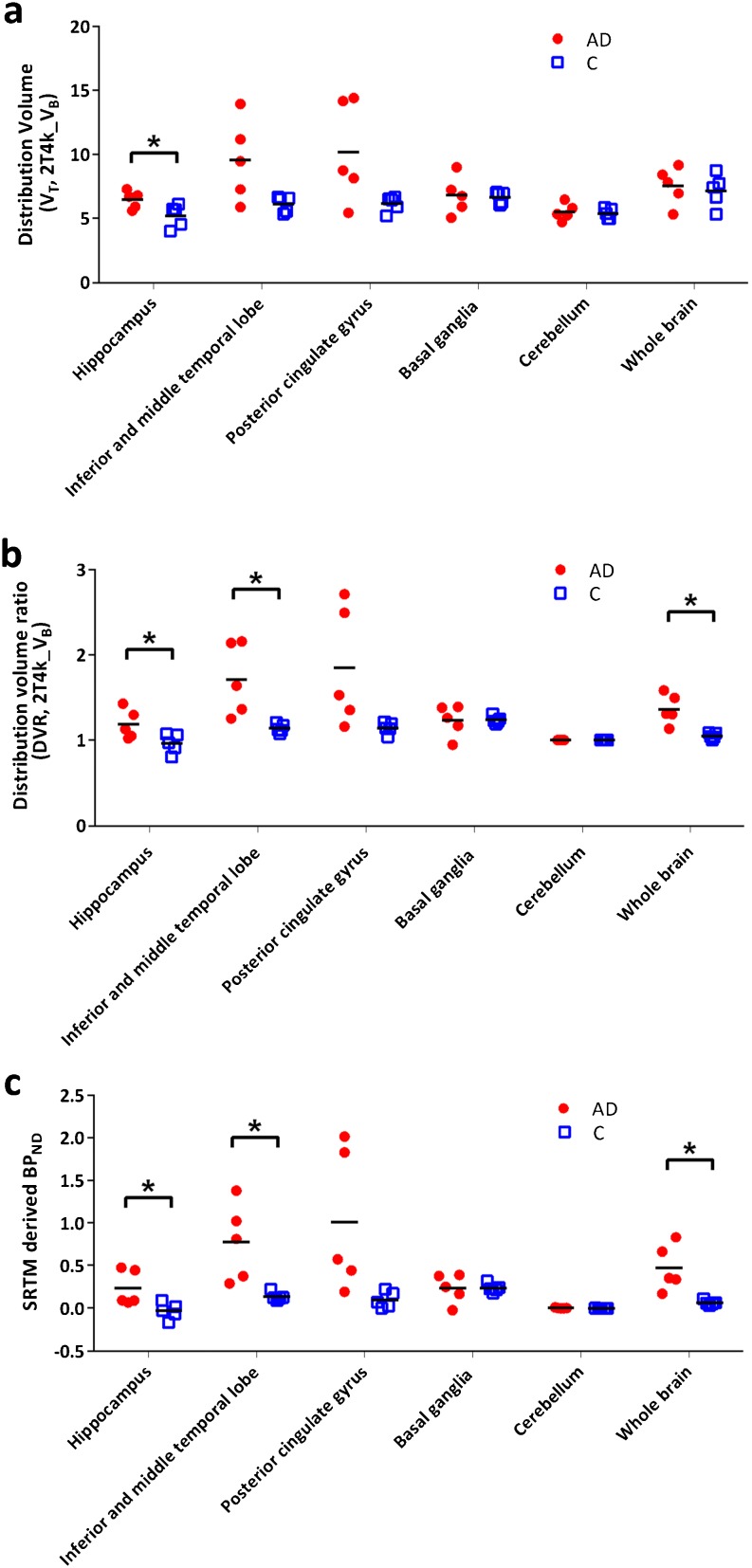
**a** V_T_, **b** DVR and **c** SRTM-derived BP_ND_ for several regions of interest (grey matter) for both AD patients and controls. *Asterisk* indicates a significant difference (*p* < 0.05) between the subject groups obtained using simple unpaired *t* test considering unequal variance.

A preliminary comparison was performed for a few tau-specific regions to assess the presence of possible differences in [^18^F]AV1451 binding between AD and normal subjects. Furthermore, possible group differences for grey matter cerebellum and whole brain grey matter were also assessed. Differences in tracer binding between subject groups were observed using either V_T_, DVR or SRTM-derived BP_ND_. A clear illustration of these differences is shown in Fig. 7.

## Discussion

A reversible two tissue compartmental model with blood volume parameter was able to properly describe tissue kinetics of [^18^F]AV1451, independent of subject status and underlying tau load. In this relatively small study population, no significant difference in grey matter cerebellum V_T_ was seen between controls and AD patients, indicating that grey matter cerebellum might be a viable reference region. Plasma input-derived BP_ND_ did not correlate with DVR-1 values, indicating limitations in the robust estimation of k_3_/k_4_, especially in controls where the second compartment was essentially absent. [^18^F]AV1451 binding appears to be different between AD patients and controls, particularly in tau-specific regions. Although further data are needed, this suggest that [^18^F]AV1451 may be useful for monitoring of disease progression or treatment effects.

Model preference seemed to be influenced by subject status or rather by the underlying tau load. Tissue kinetics in all AD subjects was best described using a 2T4k_V_B_ model, but for controls, the simpler 1T2k_V_B_ model was better. As this would complicate group comparisons, the effect of model choice on quantitative results was evaluated. This comparison showed a strong correlation between 2T4k_V_B_ and 1T2k_V_B_ derived V_T_ in controls, indicating that the 2T4k_V_B_ model still provides reliable V_T_ estimates. Although a reasonable correlation between V_T_ values from both models was obtained in AD patients, there was increasing underestimation in 1T2k_V_B_ derived V_T_ with increasing V_T_, illustrating an increasing effect of the second (tau) compartment. Based on the good correlation in controls, however, it seems safe to use the 2T4k_V_B_ model independent of subject status or tau load.

As the model preferences seem to differ between AD and C, the question arises whether it could differentiate between amyloid positive and negative scans. In the present study, two controls with an amyloid positive scan were included, but their kinetics were best described by the 1T2k_V_B_ model. In addition, in one control, tracer kinetics were best described using the 2T4k_V_B_ model, but this control had an amyloid-negative scan. Therefore, at least for the healthy controls in this study, model preference was not able to differentiate between amyloid positive and negative scan. However, further studies with a larger dataset will be needed to substantiate these findings.

Recently, Barret et al. [24] performed kinetic modelling of [^18^F]AV1451 in AD patients, using a metabolite-corrected plasma input function, and reported similar model preferences as in this study. Another very recent study [25], evaluating *in vivo* kinetics of [^18^F]AV1451 in subjects with mild cognitive impairment and a history of traumatic brain injury, also found that model preference was dependent on underlying V_T_. Similarly, a study [26] using a different tau tracer, [^18^F]THK5117, reported a similar kinetic model preference for describing tracer kinetics. In [^18^F]AV1451 studies published previously [27, 28], grey matter cerebellum was used as reference region. In the present study, no significant difference in estimated V_T_ was observed between subject groups, indicating that, at least in the present patient population, there was no tau-specific tracer uptake in cerebellum, thus supporting the use of grey matter cerebellum as reference region.

V_T_ is a measure of both specific and non-specific binding. Hence, BP_ND_ is a better estimate as it only incorporates specific binding. Since a robust estimation of k_3_/k_4_ parameter was not possible, a reliable measure of tau binding in the presence of a reference region is DVR-1. Therefore, the impact of model preference on DVR was also investigated, and again, similar results were obtained for 2T4k_V_B_ and 1T2k_V_B_ models, confirming that that 2T4k_V_B_ can be used for reliable estimation of DVR in both AD patients and controls. Furthermore, SRTM-derived BP_ND_ for all grey matter ROIs correlated well with DVR, irrespective of subject status. Therefore, although a larger comparative data set is needed, the present preliminary results indicate that [^18^F]AV1451 data can be analysed without the need for arterial sampling.

To date, several [^18^F]AV1451 studies have used a simplified method, *i.e.,* SUVr obtained from a static scan acquired from 80 to 100 min after tracer injection. As both accuracy and precision of this simplified method have not been validated yet, a comparison of SUVr against DVR estimated from the 2T4K_V_B_ model and BP_ND_ derived from SRTM was performed in this study. The high correlations obtained suggest that SUVr (80–100 min) might be a simple alternative for DVR and BP_ND_ (SRTM). However, the present preliminary results are based on a small patient cohort and should be interpreted with caution. As SUVr values may be flow dependent, the present results should be substantiated in a larger study, preferably also in patients where a reduction in cerebral perfusion may be expected.

From the neuropathological literature, it is known that in AD, tau accumulates in (trans)entorhinal cortex and subsequently spreads towards the hippocampus and inferior temporal lobe and eventually into the neocortex. This pattern is known as the Braak staging [1]. Previous studies have shown strong similarities between [^18^F]AV1451 uptake patterns and Braak staging [9, 11]. Therefore, in the present study, regions of interest were selected to assess the full spectrum of Braak staging. In addition, basal ganglia was included to assess tracer uptake in regions with ‘off-target’ binding [7, 10]. In these regions, estimated macroparameters (V_T_ and DVR) were compared between subject groups. Differences using the macroparameters can be observed; however, it seems to be dependent on the region and subject of interest. In controls, no inter subject differences were observed for any of the regions, whereas in AD subjects a higher and more variable values for V_T_ and DVR were obtained, possibly representing a tau-specific signal.

In line with previous studies [24, 27, 28], kinetics in the off-target binding regions were evaluated. Kinetics in putamen, pallidum and thalamus were different from those in other cortical regions. The model preference for these regions was 1T2k_V_B_, irrespective of subject status. In a recent study [24], a higher k_4_ was observed for putamen, pallidum and thalamus. This could be a possible explanation for the 1T2k_V_B_ model preference in these regions, as a higher k_4_ would make the second compartment less ‘visible’. Altered kinetics in these regions may be due to binding to another binding site. Further studies are needed to investigate this issue.

## Conclusion

*In vivo* brain kinetics of [^18^F]AV1451 are best described by a reversible two tissue compartmental model with blood volume parameter. Although initial results are promising, further studies are needed to completely define the limitations of using SRTM derived BP_ND_ and/or SUVr in clinical research and, thus, whether arterial sampling can be omitted. As flow sensitivity of SUVr was not evaluated, the impact of flow changes on the SUVr use in therapeutic intervention studies also needs further evaluation.

## Electronic supplementary material

ESM 1 (PDF 443 kb).
