# Supplementary material for: Quantification of Tau Load Using [18F]AV1451 PET
Source: Mol Imaging Biol. 2017 Apr 3;19(6):963–71. doi: 10.1007/s11307-017-1080-z (PMC5662681; doi:10.1007/s11307-017-1080-z)
Supplement: Supplementary file 1 — (PDF 443 kb). [file 11307_2017_1080_MOESM1_ESM.pdf]

Electronic Supplementary Material

Quantification of Tau Load using $[^{18}F]$AV1451 PET

Journal: Molecular Imaging and Biology

Sandeep SV Golla[1*#], Tessa Timmers[1,2*], Rik Ossenkoppele[1,2], Colin Groot[1], Sander Verfaillie[2], Philip Scheltens[2], Wiesje M van der Flier[2,4], Lothar Schwarte[3], Mark A Mintun[5], Michael Devous[5], Robert C Schuit[1], Albert D Windhorst[1], Adriaan A Lammertsma[1], Ronald Boellaard[1,6], Bart NM van Berckel[1,2], Maqsood Yaqub[1]

[1]Department of Radiology & Nuclear Medicine, [2]Alzheimer Center & Department of Neurology, [3]Department of Anaesthesiology, [4]Department of Epidemiology & Biostatistics, VU University Medical Center, Amsterdam, Netherlands,

[5]Avid Radiopharmaceuticals, Inc., United States,

[6]Department of Nuclear Medicine & Molecular Imaging, University of Groningen, University Medical Center Groningen, Groningen, The Netherlands.

*Both authors contributed equally to this work

#Corresponding author:

Sandeep SV Golla, MSc

Department of Radiology & Nuclear Medicine, VU University Medical Center

PO Box 7057, 1007MB Amsterdam,

The Netherlands

Tel: +31(0)611767523

E-mail: s.golla@vumc.nl

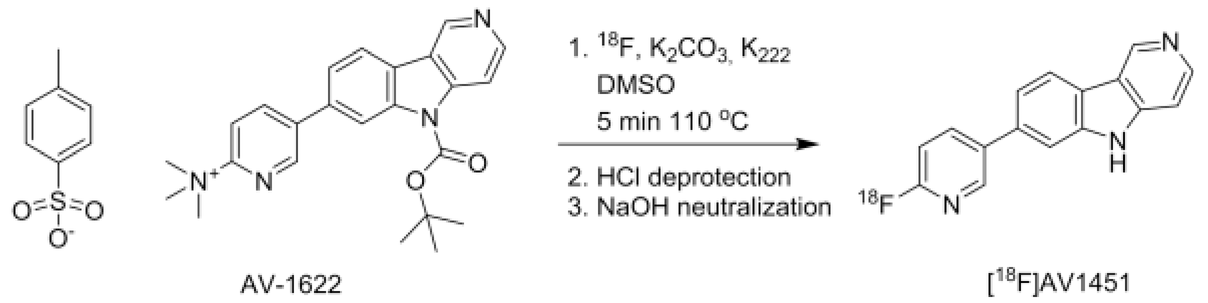

Suppl. Fig. 1. Radiosynthesis of [^{18}F]AV145.
